# Supplementary material for: Oriented Deep Eutectic Solvents as Efficient Approach for Selective Extraction of Bioactive Saponins from Husks of Xanthoceras sorbifolia Bunge
Source: Antioxidants (Basel). 2022 Apr 8;11(4):736. doi: 10.3390/antiox11040736 (PMC9026909; doi:10.3390/antiox11040736)
Supplement: Supplementary file 1 [file antioxidants-11-00736-s001.zip › antioxidants-1644102-supplementary.pdf]

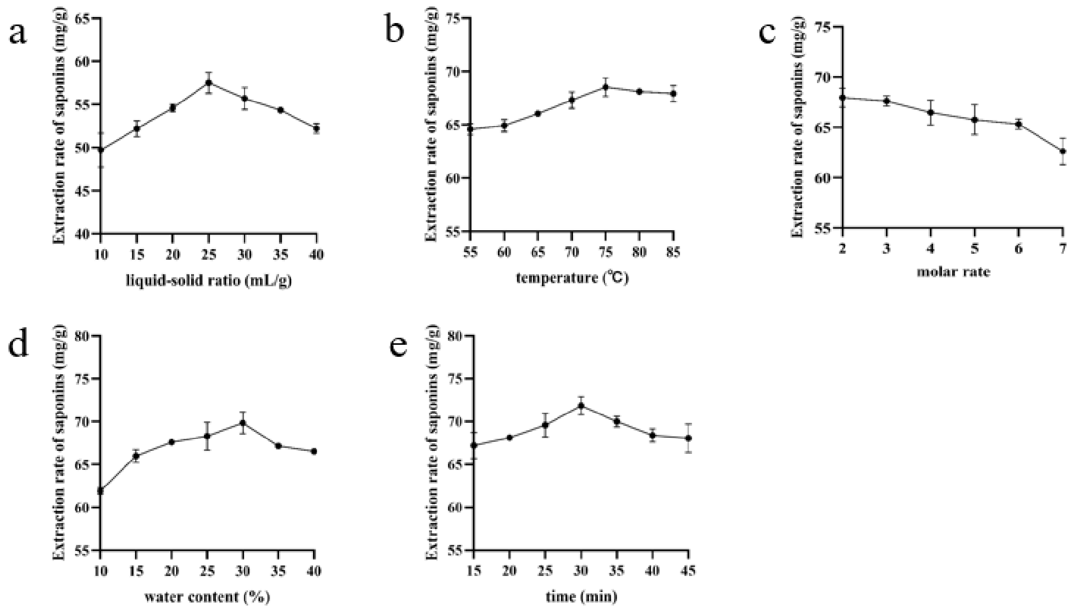

Figure S1. Five independent variables were determined for the preliminary range by single factor experiment. The range of variables and parameters is as follows: liquid-solid ratio (10, 15, 20, 25, 30, 35, and 40 mL/g), water content (10, 15, 20, 25, 30, 35, and 40 %), extraction temperature (55, 60, 65, 70, 75, 80, and 85 °C), extraction time (15, 20, 25, 30, 35, 40, and 45 min), and molar rate (1:2, 1:3, 1:4, 1:5, 1:6, 1:7) were determined for the preliminary range.

Table S1. The experimental orders, levels of variables, and response values in Box-Behnken design.

Run	Extraction Time (A, min)	Extraction Temperature (B,°C)	Liquid-Solid Ratio (C, mL/g)	Water Content (D, %)	Total Extraction Yields of Saponins (mg Re/g dw)
1	30	75	20	40	64.66±0.76
2	30	75	25	35	68.84±1.05
3	30	75	30	40	67.23±1.89
4	30	75	25	35	72.84±1.35
5	25	70	25	35	63.15±0.24
6	30	75	20	30	66.97±1.76
7	35	70	25	35	64.04±1.74
8	25	80	25	35	70.00±1.03
9	35	80	25	35	69.73±1.70

10	30	75	30	30	68.72±2.01
11	30	80	30	35	70.43±3.20
12	25	75	25	30	70.80±1.05
13	35	75	25	40	68.66±1.64
14	30	75	25	35	72.85±0.85
15	30	70	20	35	64.05±0.99
16	30	75	25	35	70.53±1.67
17	25	75	25	40	62.79±1.41
18	30	70	30	35	65.63±1.53
19	35	75	25	30	69.82±1.83
20	30	80	20	35	70.03±0.12
21	30	75	25	35	72.81±1.10
22	25	75	30	35	71.57±0.96
23	35	75	30	35	69.58±2.53
24	30	70	25	30	64.69±1.35
25	25	75	20	35	71.40±2.08
26	30	80	25	40	66.30±0.21
27	30	75	25	35	72.80±1.11
28	30	70	25	40	64.21±1.35
29	30	80	25	30	67.01±2.03
30	35	75	20	35	66.11±0.22

Table S2. Characterization of chemical compounds in TPMBr-La extracts from *X. sorbifolia* shells powder by HPLC-ESI-MS in positive mode.

No.	Rt (min)	Molecular weight (Da)	Measured mass(m/z) [M+H]+	Measured mass(m/z) [M+Na]+	MS/MS (m/z)	Molecular formula	Proposed compound
1	25.21	678	679.50	701.50	495.21	$C_{38}H_{62}O_{10}$	16-O-acetyl-21-O-*α*-L-rhamnopyranosyl-*β*-barringtogenol C
2	32.63	944	945.55	967.53	821.76<br>331.67	$C_{48}H_{80}O_{18}$	3-O-*β*-D-glucopyranosyl,28-O-[*α*-L-rhamnol(1→2)]-*β*-D-glucopyranosyl-16-deoxybarringtogenol C
3	37.34	1188	1189.65	1211.65	1211.63<br>787.28<br>447.91	$C_{59}H_{96}O_{24}$	3-O-[*β*-D-glucopyranosyl (1→6)] (3'-O-angeloyl)-*β*-D-glucopyranosyl-28-O-[*α*-L-rhamnosyl (1→2)]- *β*-D-glucopyranosyl-16-deoxybarringtogenol C
4	38.21	1392	1393.69	1415.69	1415. 69<br>948.34<br>331.34	$C_{67}H_{10}8O_{30}$	3-O-(3-O-angeloyl-4-O-acetyl-6-O-*β*-D-glucopyranosyl)-*β*-D-glucopyranosyl- 28-O-(2-*α*-L-rhamnopyranosyl-6-O-*β*-

							Dglucopyranosyl)-*β*-D-glucopyranosyl-16-deoxybarringtogenol C
5	50.90	1142	1143.58	1165.58	695.85<br>595.27<br>493.89<br>342.93	$C_{57}H_{90}O_{23}$	3-*O*-[*β*-D-galactopyranosyl-(1→2)]-*α*-L-arabinofuranosyl-(1→3)-*β*-D-glucuronopyranosyl-21-*O*-angeloyl-28-*O*-2-methylbutanoyl-3*β*,15*α*,16*α*,21*β*,22*α*,28-hexahydroxyolean-12-ene (xanifolia Y7)
6	54.20	1140	1141.57	1163.57	693.84<br>593.28<br>493.89<br>342.93	$C_{57}H_{88}O_{23}$	3-*O*-(*α*-L-arabinofuranosyl(1→3)-*β*-D-galactopyranosyl (1→2))*β*-D-glucuronopyranosyl-21,22-diangeloyl-R1-barrigenol (xanthoceracide)
7	55.26	1156	1157.57	1179.57	709.87<br>509.87<br>493.89<br>342.93	$C_{57}H_{88}O_{24}$	3-*O*-[*β*-D-glucopyranosyl-(1→2)]-*α*-L-arabinofuranosyl (1→3)-*β*-D-glucuronopyranosyl-21,22-*O*-diangeloyl-3*β*,15*α*,16*α*,21*β*,22*α*,24*β*,28-heptahydroxyolean-12-ene (xanifolia Y2)
